# Supplementary material for: Students’ experience of interpersonal interactions quality in e-Learning: A qualitative research
Source: PLoS One. 2024 Mar 26;19(3):e0298079. doi: 10.1371/journal.pone.0298079 (PMC10965068; doi:10.1371/journal.pone.0298079)
Supplement: Attachment — Submitted filename: PONE-D-23-23929_reviewer (1).pdf [file pone.0298079.s002.pdf]

# PLOS ONE

## Students' Experience of Interpersonal Interactions Quality in E-Learning: A Qualitative Research

--Manuscript Draft--

| | |
|---|---|
| **Manuscript Number:** | PONE-D-23-23929 |
| **Article Type:** | Research Article |
| **Full Title:** | Students' Experience of Interpersonal Interactions Quality in E-Learning: A Qualitative Research |
| **Short Title:** | Students' Experience of Interpersonal Interactions Quality in E-Learning |
| **Corresponding Author:** | Shirin Hasanvand, Ph.D<br>Lorestan University of Medical Sciences<br>Khorramabad, IRAN, ISLAMIC REPUBLIC OF |
| **Keywords:** | Interpersonal interactions; education; Distance; Student; Content analysis |
| **Abstract:** | Introduction<br><br>Online interaction is an essential element in distance learning, and effective online communication models empower students. This research was conducted to explain students' experiences on the quality of interpersonal interactions in e-learning.<br><br>Materials and Methods<br><br>The qualitative descriptive design via conventional content analysis was used. Sixteen participants were selected by purposeful and maximum variation method and recruited from three universities of Medical Sciences in Iran. The data were collected through semi-structured in-depth, face-to-face, or electronic interviews. Interviews were recorded through a digital recorder, and analysis was performed simultaneously with data collection using Graneheim and Lundman (2004). The Lincoln and Guba criteria, including credibility, dependability, transferability, and confirmability, were used to increase the trustworthiness of the findings.<br><br>Results<br><br>The factors that were identified were organized into three themes, identified as factors related to teaching and learning, including instructor characteristics, learners, content and subject matter, and interactions, features of the platforms consisting of asynchronous platforms, synchronous platforms, and social networks, and lastly, systemic factors including learners support.<br><br>Conclusions<br><br>To strengthen the quality of interpersonal interactions (teacher-student and student-student interactions), the role of teachers, learners, electronic content, and the system are important. Also, the characteristics of simultaneous and non-synchronous platforms, social messaging networks, and learner support are crucial. |
| **Order of Authors:** | Rita Mojtahedzadeh |
| | Shirin Hasanvand, Ph.D |
| | Aeen Mohammadi |
| | Sahar Malmir |
| | Mehdi Vatankhah |
| **Additional Information:** | |
| **Question** | **Response** |
| **Financial Disclosure** | The author(s) received no specific funding for this work. |

*Powered by Editorial Manager® and ProduXion Manager® from Aries Systems Corporation*

| | |
|---|---|
| Enter a financial disclosure statement that describes the sources of funding for the work included in this submission. Review the submission guidelines for detailed requirements. View published research articles from *PLOS ONE* for specific examples.<br><br>This statement is required for submission and **will appear in the published article** if the submission is accepted. Please make sure it is accurate.<br><br>**Unfunded studies**<br>Enter: *The author(s) received no specific funding for this work.*<br><br>**Funded studies**<br>Enter a statement with the following details:<br>• Initials of the authors who received each award<br>• Grant numbers awarded to each author<br>• The full name of each funder<br>• URL of each funder website<br>• Did the sponsors or funders play any role in the study design, data collection and analysis, decision to publish, or preparation of the manuscript?<br>• **NO** - Include this sentence at the end of your statement: *The funders had no role in study design, data collection and analysis, decision to publish, or preparation of the manuscript.*<br>• **YES** - Specify the role(s) played.<br><br>* typeset | |
| **Competing Interests**<br><br>Use the instructions below to enter a competing interest statement for this submission. On behalf of all authors, disclose any competing interests that could be perceived to bias this work—acknowledging all financial support and any other relevant financial or non-financial competing interests.<br><br>This statement is required for submission and **will appear in the published article** if | The author(s) received no specific funding for this work. |

| | |
|---|---|
| the submission is accepted. Please make sure it is accurate and that any funding sources listed in your Funding Information later in the submission form are also declared in your Financial Disclosure statement.<br><br>View published research articles from *PLOS ONE* for specific examples.<br><br>**NO authors have competing interests**<br><br>Enter: *The authors have declared that no competing interests exist.*<br><br>**Authors with competing interests**<br><br>Enter competing interest details beginning with this statement:<br><br>*I have read the journal's policy and the authors of this manuscript have the following competing interests: [insert competing interests here]*<br><br>* typeset | |
| **Ethics Statement**<br><br>Enter an ethics statement for this submission. This statement is required if the study involved:<br><br>• Human participants<br>• Human specimens or tissue<br>• Vertebrate animals or cephalopods<br>• Vertebrate embryos or tissues<br>• Field research<br><br>Write "N/A" if the submission does not require an ethics statement.<br><br>General guidance is provided below. Consult the submission guidelines for detailed instructions. **Make sure that all information entered here is included in the Methods section of the manuscript.** | This research was done under the Declarationration of Helsinki. The ethics code was obtained from the Deputy for Research and Technology of Tehran University of Medical Sciences (IR.TUMS.MEDICINE.REC.1400.686). After providing explanations about the study's objectives, written informed consent was obtained from all participants. |

| | |
|---|---|
| **Format for specific study types**<br><br>**Human Subject Research (involving human participants and/or tissue)**<br>• Give the name of the institutional review board or ethics committee that approved the study<br>• Include the approval number and/or a statement indicating approval of this research<br>• Indicate the form of consent obtained (written/oral) or the reason that consent was not obtained (e.g. the data were analyzed anonymously)<br><br>**Animal Research (involving vertebrate animals, embryos or tissues)**<br>• Provide the name of the Institutional Animal Care and Use Committee (IACUC) or other relevant ethics board that reviewed the study protocol, and indicate whether they approved this research or granted a formal waiver of ethical approval<br>• Include an approval number if one was obtained<br>• If the study involved *non-human primates*, add *additional details* about animal welfare and steps taken to ameliorate suffering<br>• If anesthesia, euthanasia, or any kind of animal sacrifice is part of the study, include briefly which substances and/or methods were applied<br><br>**Field Research**<br><br>Include the following details if this study involves the collection of plant, animal, or other materials from a natural setting:<br>• Field permit number<br>• Name of the institution or relevant body that granted permission | |
| **Data Availability**<br><br>Authors are required to make all data underlying the findings described fully available, without restriction, and from the time of publication. PLOS allows rare exceptions to address legal and ethical concerns. See the PLOS Data Policy and FAQ for detailed information. | Yes - all data are fully available without restriction |

| | |
|---|---|
| A Data Availability Statement describing where the data can be found is required at submission. Your answers to this question constitute the Data Availability Statement and **will be published in the article**, if accepted.<br><br>**Important:** Stating 'data available on request from the author' is not sufficient. If your data are only available upon request, select 'No' for the first question and explain your exceptional situation in the text box.<br><br>Do the authors confirm that all data underlying the findings described in their manuscript are fully available without restriction? | |
| **Describe where the data may be found in full sentences. If you are copying our sample text, replace any instances of XXX with the appropriate details.**<br><br>• If the data are **held or will be held in a public repository**, include URLs, accession numbers or DOIs. If this information will only be available after acceptance, indicate this by ticking the box below. For example: *All XXX files are available from the XXX database (accession number(s) XXX, XXX.*).<br>• If the data are all contained **within the manuscript and/or Supporting Information files**, enter the following: *All relevant data are within the manuscript and its Supporting Information files.*<br>• If neither of these applies but you are able to provide **details of access elsewhere**, with or without limitations, please do so. For example:<br><br>*Data cannot be shared publicly because of [XXX]. Data are available from the XXX Institutional Data Access / Ethics Committee (contact via XXX) for researchers who meet the criteria for access to confidential data.*<br><br>*The data underlying the results presented in the study are available from (include the name of the third party* | All data files are available from the figshare database. |

| | |
|---|---|
| *and contact information or URL).*<br>• This text is appropriate if the data are owned by a third party and authors do not have permission to share the data.<br><br>* typeset | |
| Additional data availability information: | Tick here if the URLs/accession numbers/DOIs will be available only after acceptance of the manuscript for publication so that we can ensure their inclusion before publication. |

Powered by Editorial Manager® and ProduXion Manager® from Aries Systems Corporation

Manuscript

Click here to access/download;Manuscript;manuscript 30.04.1402.docx

# Students' Experience of Interpersonal Interactions Quality in E-Learning: A Qualitative Research

Rita Mojtahedzadeh [1¶], Shirin Hasanvand [2*¶], Aeen Mohammadi [3¶], Sahar Malmir [4&], Mehdi Vatankhah [5&]

1. Associate Professor, Department of e-Learning in Medical Education, The Center of Excellence for e-Learning in Medical Education, School of Medicine, Tehran University of Medical Sciences, Tehran, Iran. Email:r_mojtahedzadeh@tums.ac.ir. 0000-0003-4560-6973

2. Associate Professor, Social Determinants of Health Research Center, Lorestan University of Medical Sciences, Khorramabad, Iran.Email:hasanvand.sh@gmail.com. 0000-0002-0243-9696

Department of e-Learning in Medical Education, Tehran University of Medical Sciences, Tehran, Iran

3. Associate Professor, Department of e-Learning in Medical Education, School of Medicine, Tehran University of Medical Sciences, Tehran, Iran. Email: aeen_mohammadi@tums.ac.ir.0000-0002-2745-3873

4. Assistant Professor, Social Determinants of Health Research Center, Lorestan University of Medical Sciences, Khorramabad, Iran. Email: saharmalmir93@gmail.com. 0000-0002-5726-7502

5. Student Research Committee, Lorestan University of Medical Sciences, Khorramabad, Iran. Email: mehdivatankhah74@gmail.com.0000-0002-5906-4688

* * Corresponding author

E.mail: hasanvand.sh1390@gmail.com

[¶] These authors contributed equally to this work.

[&] These authors also contributed equally to this work.

Shirin Hasanvand: Conceptualization, Data Curation, Formal Analysis, Investigation, Methodology, Project Administration, Supervision, Original Draft, Preparation, Writing Review & Editing.

Rita Mojtahedzadeh: Conceptualization, Data Curation, Formal Analysis, Investigation, Methodology, Project Administration, Writing Original Draft, Preparation, Writing – Review & Editing.

Aeen Mohammadi: Conceptualization, Data Curation, Formal Analysis, Investigation, Methodology, Project Administration, Writing Original Draft, Preparation, Writing – Review & Editing.

Sahar Malmir: Methodology, Original Draft, Preparation, Writing Review & Editing.

Mehdi Vatankhah : Methodology, Original Draft, Preparation, Writing Review & Editing.

# Abstract

## Introduction

Online interaction is an essential element in distance learning, and effective online communication models empower students. This research was conducted to explain students' experiences on the quality of interpersonal interactions in e-learning.

## Materials and Methods

The qualitative descriptive design via conventional content analysis was used. Sixteen participants were selected by purposeful and maximum variation method and recruited from three universities of Medical Sciences in Iran. The data were collected through semi-structured in-depth, face-to-face, or electronic interviews. Interviews were recorded through a digital recorder, and analysis was performed simultaneously with data collection using Graneheim and Lundman (2004). The Lincoln and Guba criteria, including credibility, dependability, transferability, and confirmability, were used to increase the trustworthiness of the findings.

## Results

The factors that were identified were organized into three themes, identified asʿ factors related to teaching and learning, including instructor characteristics, learners, content and subject matter, and interactions, features of the platforms consisting of asynchronous platforms, synchronous platforms, and social networks, and lastly, systemic factors including learners support.

## Conclusions

To strengthen the quality of interpersonal interactions (teacher-student and student-student interactions), the role of teachers, learners, electronic content, and the system are important. Also, the characteristics of simultaneous and non-synchronous platforms, social messaging networks, and learner support are crucial.



## Descriptors

Interpersonal interactions, Education, Distance, Student, Content analysis

# Introduction

Interaction is one of the common topics of research in e-learning(1). It is one of the fundamental elements of any learning experience and is recognized as a crucial construct in distance learning research(2). The results of Ghazi Saidi et al.'s study pointed to five essential teaching characteristics based on the student's perception, including caring, respectful, inspirational, providing good feedback, and giving excellent lectures. According to the students, interpersonal relationships were critical(3).

In the curriculum, whether a traditional or online course, interaction with students is one of the most critical responsibilities of instructors (4). Online interaction is one of the essential components of distance learning, and effective online communication models empower students. Distance learning and preventing negative experiences such as isolation and loneliness are crucial during the study period (5). Interpersonal communication between students and the teacher mainly occurs in the class during lectures. When the teacher gives a lecture, and the students listen, sending and receiving messages occur, resulting in effective communication. Effective communication is undoubtedly not only dependent on messages, but there are specific characteristics in which interpersonal communication can be effective(6).

Online communication aims the same as face-to-face communication: connection, exchange of information, and being heard and understood. Being aware of communication weaknesses in online environments can help them decide how to create timely(7). In the study by Sason et al. during the Covid-19 pandemic, students mentioned the necessity of interacting with the teacher. From the learners' perspective, the most common form of interaction was e-mail-mediated educational interaction. The minor form of interaction was social intimacy. Paying attention to the

role of interaction between the teacher and the learner and the importance of meeting the needs of different students, in distance teaching, teachers should find new ways to communicate with their students and use new communication methods suitable for synchronous and asynchronous learning environments(8).

However, students' interaction experiences in online environments differ from face-to-face classes (4). The main feature of online education is the physical distance between the teaching staff, peers, and educational institutions. Therefore different technological tools should be used to fill this gap and improve interactions (5). An online survey found that students in online classes face many problems, such as decreased motivation and comprehension, significantly decreased communication between them and the teachers, and isolation (7).

Teacher-student interaction during the teaching-learning process, in general, and especially in distance education, affects students' satisfaction, motivation, and ability to cope with learning tasks (8). Many studies have confirmed the relationship between learners' engagement and interpersonal interactions ((9-13). Other studies have also emphasized learner-teacher and learner-learner interactions in academic achievement, learning, and satisfaction(9, 10, 14). However, the quality of communication is essential. High levels of interaction do not necessarily facilitate effective learning. According to Mehall et al., interaction should be structured, purposeful, and accompanied by leadership, and the quality of interaction is more important than measuring its quantity(1). The quality of interpersonal interactions is a neglected link in e-learning. Further studies are needed focusing on the quality of interpersonal interactions in e-learning.

Only a little research has been done on the quality of interpersonal interactions. In most studies, the perspective and experiences of learners have been evaluated regarding the effectiveness of e-learning(15-18). In some studies, the focus has been on the learners' perspective on the challenges

of e-learning(19). Therefore, the present study was conducted to explain students' experiences of the quality of interpersonal interactions in e-learning.

# Materials and methods

## Design

The qualitative descriptive design via conventional content analysis was used to explore students' experiences of interpersonal interaction quality in e-learning. This type of design is usually appropriate when the existing theory or research literature about a phenomenon needs to be revised. Researchers do not use predetermined categories. Instead, it allows categories and category labels to flow from the data (20). This study was conducted from November 2021 to October 2022.

## Participants and data collection

The participants were recruited from three universities of Medical Sciences in Iran. Students of Tehran University of Medical Sciences (TUMS) and Shahid Beheshti Universities of Medical Sciences (SBMS)were selected from two virtual faculties studying electronic learning fields in medical sciences and educational technology. The Lorestan University of Medical Sciences (LUMS)students were from various disciplines of  Medical Sciences (Medicine, Nursing, Midwifery, Dentistry, …)who had online education experience during the Covid-19 era.

Deep and semi-structured interviews were conducted with 16 Persian-speaking students with a mean age of 33.9 years. The primary criteria for including the participants were willingness to participate in research, share experiences, experience with virtual education for at least one academic semester. The participants were selected by purposeful and maximum variation  in gender, age, marital status, semester, educational level,  field of study.

The first participant was a faculty member who was studying as a student in the field of e-learning in medical sciences and as a teacher and manager during the Covid-19 era, who had the experience of online teaching and managing issues related to e-learning. In the same way, based on each interview and the obtained data, the next participant to be interviewed was determined.

Before data collection, the participants received a phone explanation of the project. Informed consent was sent the day before the interview, electronically or in person, and after obtaining informed consent, the participants were interviewed. The interview time and place were arranged by agreement with the participants. The data were collected through semi-structured in-depth, face-to-face, or electronic interviews. Semistandard interviews, primarily used in health care, include several key questions that help define the research area (21). An interview guide was used (Table 1). The first interview was treated as a pilot interview to ensure the interview questions were appropriate and fit for purpose. The interview lasted 20 to 60 minutes(40 minutes on average). Interviews were recorded through a digital recorder and conducted by one of the researchers (corresponding author) with sufficient qualitative research experience. Interviewing was continued by data saturation.

## Data analysis

One of the researchers (the corresponding researcher) conducted the interviews ,and manually transcribed the audio recordings. Analysis was performed simultaneously with data collection and using Graneheim and Lundman (2004). Immediately after the interview, the text of the interview was transcribed, then typed and reread several times so that the researcher could get a whole understanding of the interview. Then the rereading of the interviews was continued to identify the meaningful and vital parts. Next, coding, subcategories, and categories of data were done to form meaningful categories.

The data analysis was conducted simultaneously by collecting data using the approach proposed by Granehim and Lundman(2004) with the following phases: 1) immediate transcription of interviews, 2) listening to them to obtain a general perception, 3) identification of significant parts and initial codes, 4) classification of similar initial codes in broader categories, and 5) determination of the hidden content in the data(Graneheim and Lundman 2004). Hence, after listening to the interviews, they were transcribed and read several times. In the next phase, the significant units were identified and coded. Subsequently, the codes were classified using a constant comparison technique, identifying differences and similarities, and subcategories were identified. Finally, the findings were compared, and categories were determined. Data analysis was carried out manually.

**Ethics Approval and the Consent to Participate**

This research was done under the Declarationration of Helsinki. The ethics code was obtained from the Deputy for Research and Technology of Tehran University of Medical Sciences (IR.TUMS.MEDICINE.REC.1400.686). After providing explanations about the study's objectives, written informed consent was obtained from all participants.

## Rigor

The results should be as authentic as possible, and each study must be assessed regarding the procedures used to create the finding(22). The Lincoln and Guba criteria, including credibility, dependability, transferability, and confirmability, were used to increase the trustworthiness of the findings. To increase credibility, we choose participants with various experiences of the phenomenon under study, with various genders and ages, educational levels, marital statuses, and fields of study. The long-term engagement of the researcher (12 months )was also considered. In order to determine dependability, the research team participated in the study process, and the findings were presented to several external observers to review the data analysis process. A clear description of the student's culture, context, and characteristics and the data collection and analysis process was provided to increase data transferability. The confirmability was accomplished by bracketing, reporting, and documenting the study phases and decisions accurately so that other researchers could track it if preferred and the audit trail could be conducted on analysis.

# Results

This study was conducted on 16 students with a mean age of 33.9 years. 55% of students were women. Most of the participants (67%) were married. Among the participants, 2 were students and faculty members simultaneously, and the rest were students. Most students were graduate students, and only 2 had undergraduate education. Table 2 displays an overview of the demographic data of participants. Data analysis showed three themes and eight categories. The main themes include factors related to teaching and learning, features of the platforms, and Systemic factors. The results of the interview are summarized in Table 3.

**Table** 1. Interview guide

| Participants' experiences regarding teacher-student interactions |
|---|
| 1- Tell me about your experience interacting with the teacher in the virtual class. |
| 2- How do you see interaction in synchronous online classes? How about asynchronous? |
| 3-According to your experience, what factors affect the quality of teacher-student interactions? |
| 4- If you have had a good or bad experience with a synchronous or asynchronous online class, can you tell me what the characteristics of such a class were? |
| 5- Tell me about your experience interacting with peers in the virtual class. |
| 6- How do you see interaction with peers in synchronous online classes? How about asynchronous? |
| 7- In your opinion, what factors affect the quality of student-student interactions? |
| 8- If you have had a good or bad experience with a synchronous or asynchronous online class, can you tell me what the characteristics of such a class were? |

**Table** 2. Characteristics of participants

| Participants (No) | Gender | Age (year) | Previous study | Marital status | University | Field of study | Semester | Degree |
|---|---|---|---|---|---|---|---|---|
| **P1** | Male | 20 | - | Single | LUMS* | Nursing | 5 | Bachelor |
| **P2** | Male | 45 | France language | Married | TUMS ** | e-Learning | 4 | MSc |
| **P3** | Male | 20 | - | Single | LUMS | Midwifery | 6 | Bachelor |
| **P4** | Male | 46 | Medical Records | Married | TUMS | e-Learning | 3 | MSc |
| **P5** | Male | 50 | Immunology | Married | TUMS | e-Learning | 4 | MSc |
| **P6** | Male | 30 | Health Information Technology | Single | SBMU*** | Medical Education Technology | 6 | MSc |
| **P7** | Male | 46 | Medical Education | Married | SBMU | e-Learning | 2 | MSc |
| **P8** | Male | 22 | Dentistry | Single | SBMU | e-Learning | 2 | MSc |
| **P9** | Male | 35 | Medical Records | Married | TUMS | e-Learning | 4 | MSc |
| **P10** | Male | 33 | Nursing | Single | TUMS | Medical Education Technology | 4 | MSc |
| **P11** | Male | 30 | Health Information Technology | Married | TUMS | e-Learning | 4 | MSc |
| **P12** | Female | 29 | Health Education and Health Promotion | Married | SUMS | e-Learning | 6 | MSc |
| **P13** | Male | 40 | Environmental Health Engineering | Married | SBMU | e-Learning | 6 | MSc |
| **P14** | Male | 40 | Radiology | Married | SBMU | e-Learning | 8 | MSc |
| **P15** | Male | 28 | Medical Education | Single | TUMS | e-Learning | 4 | PhD |
| **P16** | Male | 29 | Medical Education | Married | TUMS | e-Learning | 4 | PhD |

* Lorestan University of Medical Sciences

** Tehran University of Medical Sciences

*** Shahid Beheshti University of Medical Sciences

**Table 3. The theme, main categories, and subcategories obtained from content analysis**

| Theme | Main categories | Subcategories |
|---|---|---|
| **Factors related to teaching and learning** | Instructor characteristics | Creating a positive learning environment |
| | | Having pedagogical skills |
| | | Having charm and charisma |
| | | Having knowledge and skills in the field of ICT |
| | | Effective instructional design |
| | Learner characteristics | Homogeneity of learners |
| | | Having soft skills |
| | | being busy |
| | | Having knowledge and skills in the field of ICT |
| | | the number of learners |
| | Content and subject characteristics | Electronic content design according to standards |
| | | Logical sequence of lesson presentation |
| | | Applicability of the subject |
| | | previous familiarity with the subject |
| | Interaction | Provide appropriate feedback to learners. |
| | | Proper navigation of the Discussion forums |
| | | Providing the possibility to hear the voice and see the image and body language of the teacher |
| | | Appropriate assignment design |
| | | The right time to hold a class |
| | | Providing the possibility of face-to-face interactions |
| **Features of the platforms** | Asynchronous platforms | LMS user-friendliness |
| | | Permanent access to content in the LMS |
| | | Ability to track overall performance and progress in LMS |
| | | Absence of LMS technical problems |
| | Synchronous platforms | The possibility of share screen |
| | | Interruption and frequent system connection |
| | | The possibility of grouping learners in small classes |
| | Social Networks | Facilitate and strengthen interaction with social networks. |
| | | Informing with the help of social networks |
| **Systemic factors** | Learner support | Educational support |
| | | Technical support |

## Theme1. Factors related to teaching and learning

Learners' experiences showed that different aspects of teaching and learning, including instructor characteristics, learners, content and subject matter, and interactions, can be practical in interpersonal communication in distance education.

### Instructor characteristics

Most of the students emphasized creating a positive and motivational environment for learning. A friendly atmosphere with trust, reducing tension and anxiety, a happy and dynamic environment without discrimination and prejudice, and being an energetic teacher were among the characteristics that the students noticed. The teacher's pedagogical skills, such as academic qualification, a good expression technique, ability to class management, creating an environment for active participation, increasing learners' engagement, paying attention to the learning style of the learners, and getting feedback from the students was also an essential issue in the interaction between the teacher and the learners. Furthermore, the learners emphasized having charisma and the necessary knowledge and skills in software and practical instructional design.

*”If the teacher leaves the student-teacher relationship in the classroom and can be comfortable, I think this is the best interaction to let this student-teacher relationship turn into a friendship” (P.10).*

*”The atmosphere of the class was much more sincere. We did not feel anxious and worried in that class, and I think the interaction was effortless” (P.2).*

*”It is vital for a teacher to manage a class well ” (P.10).*

*”In online teaching, teachers use different communication tools ” (P.6).*

### Learner characteristics

Based on students' experiences, their age, social status, and other background variables influenced their interpersonal interactions in e-learning. Also, soft or non-technical skills such as time management, team working, self-regulation, communication skills, and following the rules were reported as necessary from the student's perspectives. The majority of informants emphasized the number of learners in interpersonal communication. Some participants remarked on the knowledge and skills of learners about ICT.

*" A very young person cannot establish a relationship with someone far from him in age. As a rule, there cannot be much empathy between these two people " (P.5).*

*" In groups, younger people or peers communicate better with each other " (P.13).*

*" In a virtual lesson or course, it is necessary that a person has some background or metacognitive skills " (P.4).*

*" The learner must strengthen these skills in herself/himself, such as time management, self-regulation, or metacognition skills; these can help her/him succeed in interaction " (P.4).*

**Content and subject characteristics**

The majority of informants emphasized the standards of creating electronic content. They said that the teachers in multimedia need to follow the principles of voicing and reading from the slides in a monotonous tone. This issue needs better interaction between the teacher and the students. Some teachers even read from the text and do not feel like they are in the classroom as students.

*" Sometimes, some lessons were essential, but our teacher spoke monotonously in multimedia. He either reads the text from that slide or lets someone else read it. You wanted to listen to your teacher's voice, but the voice was someone else's. You realized that the speaker of the text did not establish a connection with the subject and only read from it " (P7)*

*" Some teachers have only one voice in the audio files they upload. As if they are uploading a subject and they are reading it. You feel that it is not a classroom environment " (P3).*

Participants emphasized logical sequence and coherency between materials.

*” Teachers who go step by step are better than those who confuse many topics, and there is no connection between the topics. Teachers with continuity between their materials and go step by step are good ” (P9).*

In addition, the participants mentioned the applicability of the topic and their previous familiarity with it. In their opinion, this topic significantly impacted their relationship with the teacher.

*” The student's degree of interest in the lesson is significant, that is, to feel how necessary and practical the lesson is ” (P5).*

*” If the students had some background information about the subject the professor was teaching, their participation would be good ” (P6).*

**Interaction**

All participants emphasized the importance of receiving positive feedback based on principles, transparency, and respect, aiming to improve performance.

*” Now that I have entered the* learning management systems *(LMS) in my sixth semester, some of my assignments related to the previous semesters have no feedback, even though it has been a long time, and I have no hope anymore ” (P6).*

*” feedback should be respectful ” (p16).*

*” The teacher's feedback method is critical. How clear it is, for example, some professors write that you said no wrong ” (p9).*

Discussion forums are asynchronous communication tools widely used in Learning Management Systems, requiring the teacher's active participation. According to students, teachers should engage students with these discussion forums and follow up permanently, not temporarily. Some learners emphasized the help of learners to manage the discussions and encourage students to give fair criticism.

*” Unfortunately, there were few chat rooms with few interactions. It was because the teacher's presence was less ” (P6).*

*” Forums are excellent environments. It can be said that the most place where we could understand students' engagement with virtual education was in these forums ” (P4).*

*” The teacher can get help from other people to manage the discussion forum ” (P15).*

*” What I think is very effective in the interaction is that students must comment on each other's comments in the discussion forum. Criticize completely respectfully and morally ” (p8).*

The interesting subject that the participants emphasized was the possibility of hearing the voice and seeing the image and body language of the teacher.

*” Establishing emotional interaction happens only by hearing the teacher's voice and seeing the teacher's face ” (P5).*

*” Seeing the teacher's face has a significant impact. Because you see the mimicry of his face, you see the movements created on his face and communicate. Regardless, you understand the message better ” (P7).*

Designing appropriate assignments was also essential, and many participants mentioned it.

*” Homework should be something the student feels can be done for his benefit. Moreover, the assignments should be even personalized ” (p8).*

*” Students usually do their studies alone and do not interact with other students until they have to because of group activities ” (p4).*

Other factors affecting interpersonal interactions were the appropriate time for holding the class and providing the possibility of face-to-face interactions.

*” Unfortunately, simultaneous classes are not held at suitable times ” (P16).*

*" The face-to-face blocks, which are the first semester, make people see each other and talk to each other, and this interaction can improve " (P6).*

**Theme 2:Features of the platforms**

This category consists of asynchronous platforms, synchronous platforms, and social networks. It is described below.

**Asynchronous platforms**

Some participants argued that the best platforms for asynchronous communication, emphasizing LMS, support Persian font (the common language of Iran). It is user-friendly, and it is possible to have permanent access to the content and track its overall performance and progress. It also has minimal technical problems.

*" LMS must also be user-friendly " (P13).*

*" In the LMS we had, we had problems sending our assignments. Sometimes the assignments went one day, and one day they did not; server and font problems were very difficult " (P13).*

*" In LMS, students' activities and interactions can be observed, and their progress can be tracked " (P4).*

**Synchronous platforms**

Synchronous learning occurs in real-time. Regarding the synchronous platforms, some participants emphasized the necessity of screen sharing. Some participants expressed concern about frequent outages due to poor internet connection and communication interference. On the contrary, they mentioned the possibility of grouping learners in small classes as an opportunity for some synchronous platforms.

*" Adobe Connect was perfect, and it was possible to use all kinds of screens that supported the interaction of students with the teacher during teaching " (P13).*

*" The problem was the audio and image access; the sound was cut off and connected " (P3).*

*” I remember a good experience in the class of one of my teachers. The teacher came and formed several virtual rooms of three people and divided the students between the rooms, and each group had to discuss with each other. The teacher also entered the rooms and supervised ” (P11).*

### Social Networks

Social media was created as a platform to share information. In this study, students emphasized their essential role and the need to strengthen social networks to improve interpersonal interactions in e-learning. They believed neural networks are a powerful tool for informing and sharing information.

*” The messenger network provides the possibility of more interaction between the learners. According to the nature of the network, because it is much more accessible and manageable, it is possible to exchange messages faster in social networks ” (p5).*

*” Social networks facilitate interaction. I think it was easier to interact through social networks ” (p6).*

## Theme 3:Systemic factors

### Learner support

Support is one of the critical elements of success for online students, which in the present study was also mentioned by students as a critical element to strengthen interpersonal communication.

One of the expectations of the learners was educational support. Considering the registration and the nature of work and administrative affairs, which were all remote, they expected more guidance and support from the educational staff.

*” The presence of the virtual education staff was weak. For example, maybe the education staff is only guided in choosing a unit ” (P2).*

Technical support was another form of support mentioned by participants. According to students, they should receive sufficient technical support for synchronous and asynchronous learning platforms.

*" When LMS is used, the necessary support must be comprehensive " (p13).*

*" Even if they sell software, they support it 24 hours daily. Now, I am a poor student; sometimes I get confused, and I am looking for someone to guide me " (P10)*

Also, Some participants remarked that extracurricular activities are necessary to strengthen interpersonal communication. According to the students, extracurricular activities will stimulate interaction.

*" Scientific activities that stimulate interaction outside the classroom " (P6).*

*" One of the solutions is to create a space for them to interact outside the academic space. For example, an intellectual game, a simulation, or a game. Now, it can have an educational aspect, like gamification, or it can be just fun. They can be given an opportunity in that space, enter the game space as a team or any unique avatar and interact with each other according to the goal of the game " (P16).*

## Discussion

The results of the present study showed that students' experiences of e-learning were placed in three themes, including factors related to teaching and learning, system factors, and characteristics of platforms. Instructor and learner characteristics, content characteristics, interactions and related factors, synchronous and asynchronous platforms, and learner support were reported as factors affecting the interaction between teacher and learners and learner-learner.

The first theme was factors related to teaching and learning. Most students emphasized creating a positive, motivational, without-tension environment for learning. According to Xie and Derakhshan, a positive teacher–student relationship is recognized with empathy, caring, involvement, trust, and respect(23). Following the present results, banks have demonstrated that

teachers should strive to create positive learning environments. Such teachers create caring environments; positive behavior is the focus of classroom support, change of direction instead of reprimands will be a tool to change behavior. Learners are offered a variety of choices to reach an agreed educational goal. Teachers who create positive environments pay close attention to all environmental stimuli(24).

Chen et al. reported that educators should be trained in using social media to communicate online(25). These results reflect those of Yu, who found that online instructors' factors, including Instructors' attitude to e-learning, knowledge, skill, and interaction with learners, affect learners' satisfaction with e-learning. Also, the interaction between learners and instructors is the most important determining factor(26). Their agility and adaptability are also skills needed to design online content(25). Seifert demonstrated that learners expected their teachers to use technological tools appropriate to educational goals and content and to be aware of the benefits and how to use technology(27). So, a teaching-learning process in the online environment requires more competencies than face-to-face, particularly in technology(28).

From the characteristics of learners, the soft skills of critical learners were enumerated. This result agrees with Bergh et al.'s findings which showed that students had developed their soft skills and constructed their own identities through interaction with others, including teachers, peers, or patients (29). Unlike hard skills that deal with technical skills, soft skills are related to interpersonal skills, professional social skills, communication skills, and ethical attitudes(29). Also, in soft skills, students discussed skills such as time management, communication, commitment to learning, and teamwork. In the study by Seifert, the learners stated that instructor-student and student-student interaction in an online course increases learners' commitment to learning and success. They believed that good time management for learning is essential for success in the course and recommended that participants in an online course should complete assignments on time immediately after receiving them(27). According to learners, the design of e-content according to standards was also important. This result is consistent with those of Alsadhan et al., who found that Lack of standards is one of the factors affecting the development of multimedia e-learning systems(30).

Although feedback can become time-consuming(31), it is crucial for an online instructor. In this study, the participants emphasized this issue with incredibly constructive, timely, and respectful feedback. They considered feedback as an essential way of communication with their teacher. Feedback is how learners actively use comments and information from various sources to improve their learning (32, 33). These results match those observed in earlier studies. In a study by Seifert, Students stated that they expect the teacher to give them constructive feedback on their assignments during the course, not just at the end (27).

Another factor that influenced interpersonal interactions was the guidance of discussion forums by the teacher. All the participants wanted the active participation of the teacher. Similarly, in another study, learners mentioned this issue. They believed that when the discussion takes place in the forum, the learners would like to see the active participation of the instructor(27). Discussion and learning networks provide opportunities to search, obtain, and present information(34), but the teacher must continuously follow up with the students.

Features of the platforms are the second theme. Online learning can be organized into synchronous and asynchronous online courses (27). Asynchronous online learning does not happen in real-time but in the learner's program(35). The primary function of LMS is to provide a safe and flexible learning platform for learners. It aims to organize, read, and maintain educational materials (36). According to the participants, the user-friendliness of LMS had a significant impact on their interpersonal interactions.

Synchronous online learning happens in real time. This issue means that participants in this form of learning interact in a specific virtual environment at a given time(35). The participants' experiences regarding synchronous online platforms focused on poor Internet network connectivity and interaction disruption. These results are in line with those of previous studies. A study on medical students found that a poor internet connection is one of the most significant barriers to using online platforms (37).In the study of Goli et al., the reduction of Internet bandwidth was mentioned as an essential concern of online education(38). Also, Participants emphasized the critical role of social media in strengthening interpersonal interactions. According to Ofori et al., using social media for official academic communication stimulates an interactive learning environment, enhances social presence, and enhances learning outcomes(39). Social

media platforms are one of the easiest and most effective means of disseminating information (40). In this study, the participants repeatedly mentioned the advantage of social media in facilitating communication between the teacher-student and the students.

Systemic factors and the necessity of student support were the final themes of this study. Support is critical to online student success (33). Participants were willing to receive educational and technical support and extracurricular activities. Learning support services include information, resources, personnel, and related and interactive facilities. These resources cater to the diverse needs of students and effectively guide, assist, and promote their independent learning, collaborative learning, and holistic development(41). Student support services must be integral to the education system to meet students' needs and expectations (42). Zhao et al.'s study showed that learning support services should focus on cognitive, emotional, and managerial aspects of online learning, meeting personalized learning needs, improving service quality, and promoting online learning (41). However, in the present study, students focused on improving interpersonal interactions in educational and technical aspects and extracurricular programs.

In the present study, we conducted interviews with students; the teachers were not interviewed. Therefore, finding a whole picture of the phenomenon in question takes work. However, the strength of this study is its focus on student-teacher interactions and interactions between students, emphasizing different forms of synchronous and asynchronous communication.

## Conclusion

According to the results, it seems essential to develop a happy, dynamic, stress-free, and safe environment in the dimension of the teaching-learning process to increase interpersonal interactions. Educators should be equipped with a series of pedagogical skills to strengthen interactions. In addition, attention and focus on some non-technical or soft skills of learners is also essential. Both educators and students should have acquired enough skills and knowledge in IT. In addition to the importance of the teacher's inclusive role, the educational content must have the necessary standards and be available to the learners with a logical organization. Timely, respectful, and transparent feedback and the correct use of simultaneous and non-simultaneous communication tools and social networks are other vital issues in strengthening interpersonal

relationships and reducing the sense of social isolation. Also, the existence of even short opportunities for face-to-face interaction and extracurricular activities can be effective in strengthening virtual interactions. Finally, comprehensive and continuous support of learners increases the quality of interpersonal interactions.

## Abbreviations

-

## Declarations

### Consent for Publication

Not applicable

### Availability of Data and Materials

All data are fully available without restriction.

### Competing Interests

The authors declare that they have no competing interests.


### Funding

This research received no specific grant from public, commercial, or not-for-profit funding agencies.


### Authors' Contributions

S.H., R.M., and A.M. were responsible for the study conception/design. S.H. and M.V. performed the data collection. S.H. analyzed and interpreted the participant's data. S.H., R.M., A.M., M.V., and S.M. significantly contributed to the manuscript. All authors read and approved the final manuscript.

## Acknowledgments

The authors' gratitude goes to the participants who significantly contributed to this research.

## References

1. Mehall S. Purposeful Interpersonal Interaction in Online Learning: What Is It and How Is It Measured? Online Learning. 2020;24(1):182-204.

2. Vrasidas C, McIsaac MS. Factors influencing interaction in an online course. American journal of distance education. 1999;13(3):22-36.

3. Saidi LG, Vu P. Students’ perspective on higher educators: interpersonal relationship matters. SN Social Sciences. 2021;1:1-13.

4. Peltier JW, Schibrowsky JA, Drago W. The interdependence of the factors influencing the perceived quality of the online learning experience: A causal model. Journal of Marketing Education. 2007;29(2):140-53.

5. Vlachopoulos D, Makri A. Online communication and interaction in distance higher education: A framework study of good practice. International Review of Education. 2019;65(4):605-32.

6. Putri Anzari P, Pratiwi SS. What's Missing? How Interpersonal Communication Changes during Online Learning. Asian Journal of University Education. 2021;17(4):148-57.

7. Alawamleh M, Al-Twait LM, Al-Saht GR. The effect of online learning on communication between instructors and students during Covid-19 pandemic. Asian Education and Development Studies. 2020;11(2):380-400.

8. Sason H, Kellerman A, editors. Teacher-Student Interaction in Distance Learning in Emergency Situations. InSITE 2022: Informing Science+ IT Education Conferences; 2022.

9. Kim S, Kim D-J. Structural relationship of key factors for student satisfaction and achievement in asynchronous online learning. Sustainability. 2021;13(12):6734.

10. Muzammil M, Sutawijaya A, HARSASI M. Investigating student satisfaction in online learning: the role of student interaction and engagement in distance learning university. Turkish Online Journal of Distance Education. 2020;21(Special Issue-IODL):88-96.

11. Salta K, Paschalidou K, Tsetseri M, Koulougliotis D. Shift from a traditional to a distance learning environment during the COVID-19 pandemic: university students’ engagement and interactions. Science & Education. 2022;31(1):93-122.

12. Smith YM, Crowe AR. Nurse educator perceptions of the importance of relationship in online teaching and learning. Journal of Professional Nursing. 2017;33(1):11-9.

13. Kayode BK. Effect of communication management on distance learners’ cognitive engagement in Malaysian institutions of higher learning. International Review of Research in Open and Distributed Learning. 2018;19(4).

14. Sher A. Assessing the relationship of student-instructor and student-student interaction to student learning and satisfaction in web-based online learning environment. Journal of Interactive Online Learning. 2009;8(2).

15. Liu S. Student interaction experiences in distance learning courses: A phenomenological study. Online Journal of Distance Learning Administration. 2008;11(1):1-20.

16. Samsuri NN, Nadzri FA, Rom KBM. A study on the student's perspective on the effectiveness of using e-learning. Procedia-Social and Behavioral Sciences. 2014;123:139-44.

17. Gherheș V, Stoian CE, Fărcașiu MA, Stanici M. E-learning vs. face-to-face learning: Analyzing students' preferences and behaviors. Sustainability. 2021;13(8):4381.

18. Azis YM, Suharyati H, Susanti S. Student's experience of e-learning, learning process and perceived learning outcomes in economic math course. JHSS (Journal of Humanities and Social Studies). 2019;3(2):67-71.

19. Shafiei Sarvestani M, Mohammadi M, Afshin J, Raeisy L. Students' experiences of e-learning challenges; a phenomenological study. Interdisciplinary Journal of Virtual Learning in Medical Sciences. 2019;10(3):1-10.

20. Hsieh H-F, Shannon SE. Three approaches to qualitative content analysis. Qualitative health research. 2005;15(9):1277-88.

21. Gill P, Stewart K, Treasure E, Chadwick B. Methods of data collection in qualitative research: interviews and focus groups. British dental journal. 2008;204(6):291-5.

22. Graneheim UH, Lundman B. Qualitative content analysis in nursing research: concepts, procedures and measures to achieve trustworthiness. Nurse education today. 2004;24(2):105-12.

23. Xie F, Derakhshan A. A conceptual review of positive teacher interpersonal communication behaviors in the instructional context. Frontiers in Psychology. 2021;12:708490.

24. Banks T. Creating positive learning environments: Antecedent strategies for managing the classroom environment & student behavior. Creative Education. 2014;2014.

25. Chen X, Vorvoreanu M, Madhavan K. Mining social media data for understanding students' learning experiences. IEEE Transactions on learning technologies. 2014;7(3):246-59.

26. Yu Q. Factors influencing online learning satisfaction. Frontiers in Psychology. 2022;13.

27. Seifert T. STUDENTS'PERCEPTİONS OF ONLINE TEACHING AND LEARNING. Malaysian Online Journal of Educational Technology. 2021;9(3):1-12.

28. Bolliger DU, Halupa C. An investigation of instructors' online teaching readiness. TechTrends. 2022;66(2):185-95.

29. Bergh A-M, Van Staden C, Joubert PM, Kruger C, Pickworth GE, Roos JL, et al. Medical students' perceptions of their development of'soft skills': part II: the development of'soft skills' through'guiding and growing'. South African Family Practice. 2006;48(8):1.

30. Alsadhan AO, Alhomod S, Shafi MM. Multimedia based E-learning: Design and integration of multimedia content in E-learning. International Journal of Emerging Technologies in Learning (Online). 2014;9(3):26.

31. Crespo MAJ, editor Feedback in Online Translation Courses and the Covid Era. Proceedings of the Translation and Interpreting Technology Online Conference; 2021.

32. Yang M, Mak P, Yuan R. Feedback experience of online learning during the COVID-19 pandemic: Voices from pre-service English language teachers. The Asia-Pacific Education Researcher. 2021;30(6):611-20.

33. Rotar O. Online student support: A framework for embedding support interventions into the online learning cycle. Research and Practice in Technology Enhanced Learning. 2022;17(1):1-23.

34. Emmah V. Online Discussion Forum: A Tool for Effective Student-Teacher Interaction. Available at SSRN 2525047. 2014.

35. Rigo F, Mikuš J. Asynchronous and synchronous distance learning of English as a foreign language. Media Literacy and Academic Research. 2021;4(1):89-106.

36. Xin NS, Shibghatullah AS, Abd Wahab MH, editors. A systematic review for online learning management system. Journal of Physics: Conference Series; 2021: IOP Publishing.

37. Dost S, Hossain A, Shehab M, Abdelwahed A, Al-Nusair L. Perceptions of medical students towards online teaching during the COVID-19 pandemic: a national cross-sectional survey of 2721 UK medical students. BMJ open. 2020;10(11):e042378.

38. Goli A, Chintagunta PK, Sriram S. Effects of payment on user engagement in online courses. Journal of Marketing Research. 2022;59(1):11-34.

39. Ofori S, Choongo J, Kekop M, Shah GH, Rochani H, Telfair J. Social Media Usage and Transitioning into Online Classes During COVID-19-A Survey of Undergraduate Students in Georgia, United States. International Journal of Education and Development using Information and Communication. 2021;17(4):67.

40. Sobaih AEE, Palla IA, Baquee A. Social media use in e-learning amid COVID 19 pandemic: Indian students' perspective. International Journal of Environmental Research and Public Health. 2022;19(9):5380.

41. Zhang J, Ding Y, Yang X, Zhong J, Qiu X, Zou Z, et al. COVID-19's impacts on the scope, effectiveness, and interaction characteristics of online learning: A social network analysis. Plos one. 2022;17(8):e0273016.

42. Shabani DO, Maboe K. The effectiveness and efficiency of student support services in open distance learning institutions in Africa: a desktop review. 2021.
