# Supplementary material for: Students’ experience of interpersonal interactions quality in e-Learning: A qualitative research
Source: PLoS One. 2024 Mar 26;19(3):e0298079. doi: 10.1371/journal.pone.0298079 (PMC10965068; doi:10.1371/journal.pone.0298079)
Supplement: Attachment — Submitted filename: Dear members of the editorial board.pdf [file pone.0298079.s004.pdf]

Dear members of the editorial board,

Thank you for considering me as a reviewer for the paper "Students' Experience of Interpersonal Interactions Quality in e-Learning: A Qualitative Research". It gave me the privileged to review the revised version of the paper and check for the quality of the corrections made by the authors and evaluating the manuscript's suitability for publication in *PLOS ONE*.

I provided a detailed review/evaluation referring to each comment of the previous reviewers with the correction made by the authors in the manuscript. In my overall opinion, the revised version has met the standards of the *PLOS ONE* journal and could be considered for publication.

Best regards,

Maria Shobeiry, PhD

Adjunct Assistant Professor at the University of Tehran, Department of Applied Linguistics .

Previous reviewers' comments and my evaluation of the quality of the correction:

Along with the reviewers' comments, please do consider the following points:

1. The abstract is not informative. It lacks adequate knowledge about methodology. Moreover, whatever you wrote as Conclusion are not Conclusion but Implication. Please divide the Abstract into 6 parts: Background, Purpose, Method, Results, Conclusion, Implications. It was corrected (P: 2,3)

My comment on this correction:

If the guideline of the journal accepts the corrected format, then it is ok; however I checked the authorship guideline of the PLOS ONE and I found the following:

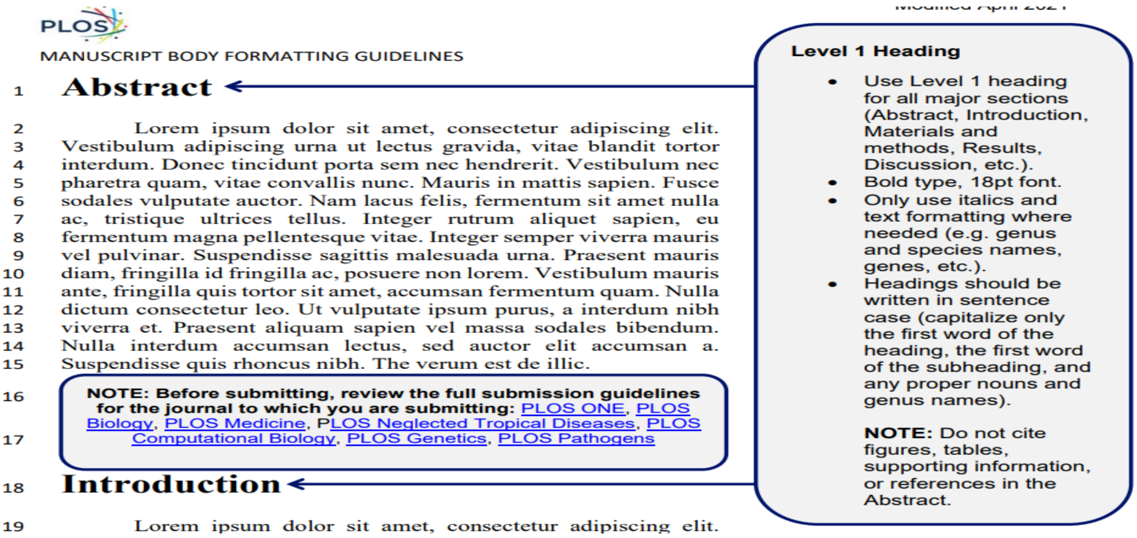
PLOS

MANUSCRIPT BODY FORMATTING GUIDELINES

Abstract

Lorem ipsum dolor sit amet, consectetur adipiscing elit. Vestibulum adipiscing urna ut lectus gravida, vitae blandit tortor interdum. Donec tincidunt porta sem nec hendrerit. Vestibulum nec pharetra quam, vitae convallis nunc. Mauris in mattis sapien. Fusce sodales vulputate auctor. Nam lacus felis, fermentum sit amet nulla ac, tristique ultrices tellus. Integer rutrum aliquet sapien, eu fermentum magna pellentesque vitae. Integer semper viverra mauris vel pulvinar. Suspendisse sagittis malesuada urna. Praesent mauris diam, fringilla id fringilla ac, posuere non lorem. Vestibulum mauris ante, fringilla quis tortor sit amet, accumsan fermentum quam. Nulla dictum consectetur leo. Ut vulputate ipsum purus, a interdum nibh viverra et. Praesent aliquam sapien vel massa sodales bibendum. Nulla interdum accumsan lectus, sed auctor elit accumsan a. Suspendisse quis rhoncus nibh. The verum est de illic.

NOTE: Before submitting, review the full submission guidelines for the journal to which you are submitting: PLOS ONE, PLOS Biology, PLOS Medicine, PLOS Neglected Tropical Diseases, PLOS Computational Biology, PLOS Genetics, PLOS Pathogens

Introduction

Lorem ipsum dolor sit amet, consectetur adipiscing elit.

Level 1 Heading

- Use Level 1 heading for all major sections (Abstract, Introduction, Materials and methods, Results, Discussion, etc.).
- Bold type, 18pt font.
- Only use italics and text formatting where needed (e.g. genus and species names, genes, etc.).
- Headings should be written in sentence case (capitalize only the first word of the heading, the first word of the subheading, and any proper nouns and genus names).

NOTE: Do not cite figures, tables, supporting information, or references in the Abstract.

Obviously in this format there is no clear separation of the six parts that the authors wrote in their revised paper. Once again, if the separated format is accepted, then it is ok.

2. The paper needs careful proofreading. It suffers from language problems, grammar, repetitions, tenses, subject-verb disagreement, etc). The whole of the paper was reworked (P:4-7).

My comment on this correction:
The quality of writing sounded ok to me. Still, in the revised version, there are some sentences which are written incohesively. But, overall, I would give it a passing score for publication as the structure of the writing does not interfere with the fluency of the text. It is ok.

3. The introduction does not build a logical case and context for the problem statement. It was corrected (P:4-7).

My comment on this correction:
The correction seemed fine to me.

4. Theoretical framework/s and empirical evidence for introducing and supporting variables are weak in this paper. The empirical evidence for supporting variables was added(P:4-7).

My comment on this correction:
The correction seemed acceptable to me.

5. The introduction needs more focus on setting the context- describe the situation followed by the 'problem' which leads to the research question and your approach to solving the problem. More importantly, The paper lacks Statement of the Problem. What is (are) the problem(s) of this study? The authors should explain it in a separate subheading. In Statement of the Problem, the authors should first mention the problems and then bring at least one national finding and one international finding related to the problems. The Introduction should provide background information and the aims and rationale behind the paper. This will allow a clear understanding of the context and importance of the study. The "big picture' relevance is important in the introduction which I couldn't see it. The statement of the problem was corrected(P:4-7).

My comment on this correction:
The statement of the problem is clearly stated in the revised version.

6. The paper needs a literature review. In addition, and most importantly, the theoretical frameworks are missed from the manuscripts. For example, how does the data shape our current understanding of the topic? There was no conceptual framework to understand the data and the data was not as developed as I would expect to see in an academic journal article.

My comment on this correction:

In the revised version, the literature is fine and, to some extent, the reader can get an overall understanding of the conceptual framework.

7. To my humble opinion, discussion needs a deeper look. In discussion: 1) the authors should mention the main reasons behind these results. Why such results obtained? What were the plausible reasons? 2) The authors should mention whether the result of their study is in line or in contrast with the previous studies by giving critical reasons. 3) Discussion must be linked to theoretical frameworks. It can be strengthened by supporting the results with theoretical framework. It may mean that the authors must tell the readers and discuss what theory/theories support their findings. The discussion was revised and modified again(P:16-21).

My comment on this correction:
The discussion of the revised version seemed fine to me.

8. Please add the implications of the study, limitations of the study, and suggestions for future researchers. They were mentioned in the discussion (P:22).

My comment on this correction:
Implications are fine now, even though it could have been written in more detail.

9. 10. Please add more updated references (2021-2023). Updated references were used and modified(P:26-8).

My comment on this correction:
The amount of the cutting-edge studies added to the revised manuscript seem fine to me.

==============================
4. Is the manuscript presented in an intelligible fashion and written in standard English?
PLOS ONE does not copyedit accepted manuscripts, so the language in submitted articles must be clear, correct, and unambiguous. Any typographical or grammatical errors should be corrected at revision, so please note any specific errors here.
Reviewer #1: Yes
Reviewer #2: Yes
Reviewer #3: Yes
Reviewer #4: No: It was corrected.

My comment on this correction:
Still, in the revised version, there are some sentences which are written incohesively. But, overall, I would give it a passing score for publication as the structure of the writing does not interfere with the fluency of the text. It is ok.

Review Comments to the Author
Reviewer #1: The present study has been done with a clear method and the findings have been well defined and discussed. Tips for improving the article have been included in the text as comments, which are provided to you in the attached text.
1.Merge the first sentence with the second sentence. The second sentence is a repetition of the first sentence but in a different form. It is better to give a specific definition of interaction in the e-Learning and tell its characteristic feature. The sentences were merged, corrections were made1. (P:4).

My comment on this correction:

A clear definition is stated in the revised version and the correction is ok to me.

2.The previous two universities have faculties or virtual education centers and are among the top rank universities of the country. What is the similarity between Lorestan University and these two? Why were this university chosen? Due to the concurrency of the study with the COVID pandemic, the sampling was first started from the selected University of Medical Sciences. Due to the sudden transition from face-to-face to virtual education and to gain richer experiences for the participants, the following students of the two virtual faculties were supposed key informants. The critical issue is that some students simultaneously studied in Medical Sciences universities as virtual colleges. In addition, the criteria for selecting participants was to complete at least one semester of training.

My comment on this correction:
The authors' answer to this comment was logical.

3.Did the first interviewee have any special characteristics that you introduced? If not, I don't think this section is necessary and it is enough to point out how the work has reached saturation. It has already been mentioned and highlighted in the text of the manuscript. The corrections of the second part of the question were also applied(P:8).

My comment on this correction:
The correction regarding the first interview could have been better but it has removed the ambiguity.

4.Teaching and learnings' Factors :The desired theme label was modified(P:10).

My comment on this correction:
In the revised manuscript the themes are unambiguous and clear.

5. This category (interaction) does not fit the rest of the categories. Also, the items in it can easily be allocated in the rest of the categories of this section ? The interaction category was removed, and based on similarity with other categories and subcategories, it was merged with them(Result and discussion section).

My comment on this correction:
Merging the subcategories made the revised version much more fluent to follow.

6.Include some sources that have conflicting findings with your work in your discussion and discuss the reasons for the contradiction with them, this will add credibility to your work. It was corrected and highlighted(P:22).

My comment on this correction:
I believe, "Zhao et al.'s ", is the study added to the revised version to attend to this comment. In my idea, the outcome of the revision is acceptable in this regard.

Reviewer #2: it seems that in the method of content analysis based on Granheim and Lundman's method, as explained in the method, codes and subcategories and category are presented, and categories are not combined and themes are not extracted. In the presented table, it seems that the first column is not the subcategory, but the extracted codes, and the second column is the subcategories and the third column is the category.
Table No. 3 shows the formation of themes, categories, and subcategories. According to the table, themes are extracted from the combination of categories. For example, categories including learners' characteristics, teachers' characteristics, content ,and subject characteristics together form the theme of factors related to teaching-learning.
Regarding ***the characteristics of the learners*** as a category, ***"creating a positive learning environment"*** is a subcategory that consists of the following codes: 1. A friendly atmosphere with trust 2. creating a happy and dynamic atmosphere 3. Being energetic 4. Non-discrimination and prejudice 5. Flexibility 6. Empathy with the student or the ***"Having pedagogical skills"*** subcategory consists of primary codes: 1. Good presentation and speaking skills 2. Class management 3. Engaging students 4. Getting feedback from students 5. Having academic competence 6. Rewarding participation

My comment on this correction:
The explanations of the authors sounded convincing to me.

System factors are mentioned and it is expected that it will have sub-layers as a layer. Based on the evidence of this study and personal experience, I would like to say that system factors can include two sub-categories, including student-related factors and teacher-related factors, and the codes related to each of these sub-categories can be educational support and support. technical support related to the student as well as educational support and technical support related to the teacher and according to experience and articles studied with the same analysis method, a category cannot end with one theme. In the interviews, the need to support the instructors has yet to be mentioned. If the professors were interviewed, according to your comment, this would be mentioned.

My comment on this correction:
The authors' answer sounded ambiguous to me. However, since the paper, in this regard, is not unclear, I believe it would be fine to move on.

In the method, it’s noted medical students, including doctors, dentists, etc., were interviewed, while only one nurse and midwife was interviewed, and the rest of the students were related to e-learning. Therefore, it is better to mention it as a limitation in generalizing the findings. Based on the results and limitations, suggestions for further studies should be provided. In qualitative research, generalizability is not considered.
However, according to the valuable comment of the honorable reviewer, sampling with maximum variation is necessary. Due to the general nature of their education (blended learning), most of the students interviewed were students of virtual colleges who often had a background in medicine-related fields. Virtualization of education in all educational institutions, students of one of the universities of medical sciences were selected as samples. The selected participants from the desired university of medical sciences included a Nursing student, a Midwifery student, and a Dental student.
On the other hand, due to the coincidence of the study with the COVID pandemic, the sampling was first started from the selected University of Medical Sciences. Due to the sudden transition from face-to-face to virtual education and to gain richer experiences for the participants, the following students of the two virtual faculties were considered key informants. The critical point is that some students simultaneously studied in medical sciences universities as virtual colleges.

My comment on this correction:
The authors' answer to this comment was clear, strong, and convincing. The paper sounded ok to me in terms of participants and sampling procedure.

---- -
Reviewer #3: A valuable manuscript on teaching and learning. The background and methods were explained well.
Abstract:
Consider using more specific terms when describing the research methods. For example, you mentioned "electronic interviews," which could be clarified as "online interviews" or "virtual interviews."
Various terms are used, such as online, digital, virtual, and e-interview. In specialized texts and articles, the term electronic interview is also standard. Please refer to the following reference. However, according to the reviewer's opinion, the word online was replaced(P:8).

My comment on this correction:
The authors have replaced the mentioned terms and the revised manuscript shows no ambiguity or inconsistency with regard to terminology.

It might be beneficial to include a brief sentence highlighting the main practical implications of your study. What can educators, institutions, or policymakers learn from your findings.

My comment on this correction:
The revised implications seem ok to me.

You've mentioned that "the quality of interpersonal interactions is a neglected link in elearning," you could expand on this by briefly discussing why this gap in research exists and why it's important to fill it. It was corrected (P: 6).

My comment on this correction:
The expansion of gap in the revised version sounds ok to me.

Carefully review the grammar, especially in longer sentences. It was corrected.

My comment on this correction:
Still, in the revised version, there are some sentences which are written incohesively. But, overall, I would give it a passing score for publication as the structure of the writing does not interfere with the fluency of the text. It is ok.

Please provide a sufficient explanation about the field of study and students in the methods section. It was added(P:7-8).

My comment on this correction:
The explanations added in this regard enriched the text and are fine.

Conclusion:
It would be good if the author could explain the practical implication of this study. It was added(P:22).

My comment on this correction:
In the revised version conclusion has been enriched and sounds ok to me.
…………………………………………………………………………………………………
Reviewer #4: The study suffers from some pitfalls. They are listed below:
- The introduction is chaotic. It does not mirror the significance of the study. It was corrected(P:4-7).

My comment on this correction:
The revised introduction is ok now. It shows the gap and is grounded on solid previous studies.

- The study lacks a rigorous literature review to explain the key concepts of the study. It was added, and highlighted(P:22).

My comment on this correction:
The revised literature shows the conceptual framework and illustrates some empirical studies. This qualifies it for an acceptable literature section for publication in my opinion. Although adding some evaluations of the previous research from a critical perspective would have made it more convincing.

- The design of the study is not clear. Explain why the authors have used this design. The study's design (qualitative research of the content analysis type) is mentioned. The reason for choosing it is explained below. However, additional clarifications were added and highlighted(P:7).

My comment on this correction:
The research design is fine; however, the reasons for choosing such a design is missing from the text. Overall it is ok now.

- The participants section needs revisions to include more demographic information about the participants. Moreover, the author should explain how they accessed the participants and earned their consent. Regarding the participants' demographic information, according to the sampling method (purposive and with maximum variation), we tried to complete the necessary information including the following: The desired theme label was modified. Gender, Age (year), Previous study, Marital status, University, Field of study, Semester, Degree. One of the crucial challenges of electronic and online interviews is ethical considerations and obtaining consent from participants. For this purpose, the informed consent form was sent to the participants one day before the interview through email or social networks so that they had enough time to read it. The interview was conducted after obtaining consent and signing and sending the consent form to the researcher. This phrase was added in the text.

My comment on this correction:
The participants and sampling procedure in the revised manuscript sounds ok to me.

- The procedures taken for data analysis should be expanded and verified. The data analysis procedures should be clearly mentioned and defined for readers. It was corrected, and highlighted(P:8-9).

My comment on this correction:
The revised version of the data analysis seems convincing.

- Enough information about the results should be provided for readers. It was corrected(P:9-16).

My comment on this correction:
The revised version of the results are clearer and enriched.

- The qualitative results need to be revised. First present the theme, then define it, after that offer some excerpts to support the themes. It was corrected (P:9-16).

My comment on this correction:
The revised version of the themes are fine to me now. It is fluent to follow and clearly explained.

- The discussion section is really poor. It does not discuss the findings critically. The discussion was revised(P:16-21).

My comment on this correction:
The revised version is much better; however, adding some elevations from a critical perspective would increase the quality of the paper.

- The study does not have suggestions for further research. It was added(P:21).

My comment on this correction:
In my humble opinion, the suggestions for further studies is very poor and is confined only to suggestions for conducting in various contexts. I believe it is fine for meeting a passing score for publication but it is definitely not enough!

- The study lacks implications for the pertinent stakeholders. Please fix this problem. It was fixed(P:22).

My comment on this correction:
The revised version is fine with regard to implications.

- The references are not following APA guidelines. Please revise them. PLOS uses "Vancouver" style, as outlined in the ICMJE sample references. (Guideline for authors)
With best regards,

My comment on this correction:

Vancouver style has been employed in the revised version.
