# Supplementary material for: Students’ experience of interpersonal interactions quality in e-Learning: A qualitative research
Source: PLoS One. 2024 Mar 26;19(3):e0298079. doi: 10.1371/journal.pone.0298079 (PMC10965068; doi:10.1371/journal.pone.0298079)
Supplement: Attachment — Submitted filename: Response to Reviewers (2).docx [file pone.0298079.s003.docx]

Dear authors,

Thank you for submitting your manuscript to PLOSE ONE.

I have now received the reviews from the reviewers. After careful consideration, we feel that it has merit but needs revision. Please carefully address the comments and provide a line-by-line letter of response and highlight all the changes you make with different comments. If you disagree with the reviewers' comments, please write a rebuttal justifying your disagreement.

Along with the reviewers' comments, please do consider the following points:

Thanks for your valuable comments

1. The abstract is not informative. It lacks adequate knowledge about methodology. Moreover, whatever you wrote as Conclusion are not Conclusion but Implication. Please divide the Abstract into 6 parts: Background, Purpose, Method, Results, Conclusion, Implications. It was corrected (P: 2,3)
2. The paper needs careful proofreading. It suffers from language problems, grammar, repetitions, tenses, subject-verb disagreement, etc). The whole of the paper was reworked (P:4-7).
3. The introduction does not build a logical case and context for the problem statement. It was corrected(P:4-7).
4. Theoretical framework/s and empirical evidence for introducing and supporting variables are weak in this paper. The empirical evidence for supporting variables was added(P:4-7).
5. The introduction needs more focus on setting the context- describe the situation followed by the ‘problem’ which leads to the research question and your approach to solving the problem. More importantly, The paper lacks Statement of the Problem. What is (are) the problem(s) of this study? The authors should explain it in a separate subheading. In Statement of the Problem, the authors should first mention the problems and then bring at least one national finding and one international finding related to the problems. The Introduction should provide background information and the aims and rationale behind the paper. This will allow a clear understanding of the context and importance of the study. The “big picture’ relevance is important in the introduction which I couldn’t see it. The statement of the problem was corrected(P:4-7).
6. The paper needs a literature review. In addition, and most importantly, the theoretical frameworks are missed from the manuscripts. For example, how does the data shape our current understanding of the topic? There was no conceptual framework to understand the data and the data was not as developed as I would expect to see in an academic journal article. It was corrected(P:4-7).
7. To my humble opinion, discussion needs a deeper look.  In discussion: 1) the authors should mention the main reasons behind these results. Why such results obtained? What were the plausible reasons? 2) The authors should mention whether the result of their study is in line or in contrast with the previous studies by giving critical  reasons. 3) Discussion must be linked to theoretical frameworks. It can be strengthened by supporting the results with theoretical framework. It may mean that the authors must tell the readers and discuss what theory/theories support their findings. The discussion was revised and modified again(P:16-21).
8. Please add the implications of the study, limitations of the study, and suggestions for future researchers. They were mentioned in the discussion(P:22).
9. 10. Please add more updated references (2021-2023). Updated references were used and modified(P:26-8).

Good Luck

Ehsan Namaziandost

Academic Editor
==============================

Please submit your revised manuscript by Oct 29 2023 11:59PM. If you will need more time than this to complete your revisions, please reply to this message or contact the journal office at [plosone@plos.org](mailto:plosone@plos.org). When you're ready to submit your revision, log on to <https://www.editorialmanager.com/pone/> and select the 'Submissions Needing Revision' folder to locate your manuscript file.

Please include the following items when submitting your revised manuscript:

- A rebuttal letter that responds to each point raised by the academic editor and reviewer(s). You should upload this letter as a separate file labeled 'Response to Reviewers'.
- A marked-up copy of your manuscript that highlights changes made to the original version. You should upload this as a separate file labeled 'Revised Manuscript with Track Changes'.
- An unmarked version of your revised paper without tracked changes. You should upload this as a separate file labeled 'Manuscript'.

If you would like to make changes to your financial disclosure, please include your updated statement in your cover letter. Guidelines for resubmitting your figure files are available below the reviewer comments at the end of this letter.

If applicable, we recommend that you deposit your laboratory protocols in [protocols.io](http://protocols.io/) to enhance the reproducibility of your results. Protocols.io assigns your protocol its own identifier (DOI) so that it can be cited independently in the future. For instructions see: <https://journals.plos.org/plosone/s/submission-guidelines#loc-laboratory-protocols>. Additionally, PLOS ONE offers an option for publishing peer-reviewed Lab Protocol articles, which describe protocols hosted on [protocols.io](http://protocols.io/). Read more information on sharing protocols at <https://plos.org/protocols?utm_medium=editorial-email&utm_source=authorletters&utm_campaign=protocols>.

We look forward to receiving your revised manuscript.

Kind regards,

Ehsan Namaziandost
Academic Editor
PLOS ONE

**Comments to the Author**

Thanks for reviewer’s valuable comments

1. Is the manuscript technically sound, and do the data support the conclusions?

The manuscript must describe a technically sound piece of scientific research with data that supports the conclusions. Experiments must have been conducted rigorously, with appropriate controls, replication, and sample sizes. The conclusions must be drawn appropriately based on the data presented.

Reviewer #1: Yes

Reviewer #2: Yes

Reviewer #3: Yes

Reviewer #4: Partly

2. Has the statistical analysis been performed appropriately and rigorously?

Reviewer #1: Yes

Reviewer #2: Yes

Reviewer #3: Yes

Reviewer #4: Yes

3. Have the authors made all data underlying the findings in their manuscript fully available?

The [PLOS Data policy](http://www.plosone.org/static/policies.action#sharing) requires authors to make all data underlying the findings described in their manuscript fully available without restriction, with rare exception (please refer to the Data Availability Statement in the manuscript PDF file). The data should be provided as part of the manuscript or its supporting information, or deposited to a public repository. For example, in addition to summary statistics, the data points behind means, medians and variance measures should be available. If there are restrictions on publicly sharing data—e.g. participant privacy or use of data from a third party—those must be specified.

Reviewer #1: Yes

Reviewer #2: Yes

Reviewer #3: Yes

Reviewer #4: No : All data underlying the findings in the manuscript fully available

4. Is the manuscript presented in an intelligible fashion and written in standard English?

PLOS ONE does not copyedit accepted manuscripts, so the language in submitted articles must be clear, correct, and unambiguous. Any typographical or grammatical errors should be corrected at revision, so please note any specific errors here.

Reviewer #1: Yes

Reviewer #2: Yes

Reviewer #3: Yes

Reviewer #4: No: It was corrected.

5. Review Comments to the Author

Reviewer #1: The present study has been done with a clear method and the findings have been well defined and discussed. Tips for improving the article have been included in the text as comments, which are provided to you in the attached text.

1.Merge the first sentence with the second sentence. The second sentence is a repetition of the first sentence but in a different form. It is better to give a specific definition of interaction in the e-Learning and tell its characteristic feature. The sentences were merged, corrections were made1.	(P:4).

2.The previous two universities have faculties or virtual education centers and are among the top rank universities of the country. What is the similarity between Lorestan University and these two? Why were this university chosen? Due to the concurrency of the study with the COVID pandemic, the sampling was first started from the selected University of Medical Sciences. Due to the sudden transition from face-to-face to virtual education and to gain richer experiences for the participants, the following students of the two virtual faculties were supposed key informants. The critical issue is that some students simultaneously studied in Medical Sciences universities as virtual colleges. In addition, the criteria for selecting participants was to complete at least one semester of training.

3.Did the first interviewee have any special characteristics that you introduced? If not, I don't think this section is necessary and it is enough to point out how the work has reached saturation. It has already been mentioned and highlighted in the text of the manuscript. The corrections of the second part of the question were also applied(P:8).

4.Teaching and learnings' Factors: The desired theme label was modified(P:10).

5. This category ( interaction) does not fit the rest of the categories. Also, the items in it can easily be allocated in the rest of the categories of this section ? The interaction category was removed, and based on similarity with other categories and subcategories, it was merged with them(Result and discussion section).

6.Include some sources that have conflicting findings with your work in your discussion and discuss the reasons for the contradiction with them, this will add credibility to your work. It was corrected and highlighted(P:22).

------------------------------------------------------------------------------------------------

Reviewer #2: it seems that in the method of content analysis based on Granheim and Lundman's method, as explained in the method, codes and subcategories and category are presented, and categories are not combined and themes are not extracted. In the presented table, it seems that the first column is not the subcategory, but the extracted codes, and the second column is the subcategories and the third column is the category.

Table No. 3 shows the formation of themes, categories, and subcategories. According to the table, themes are extracted from the combination of categories. For example, categories including learners' characteristics, teachers' characteristics, content ,and subject characteristics together form the theme of factors related to teaching-learning. Regarding ***the characteristics of the learners*** as a category, ***"creating a positive learning environment"*** is a subcategory that consists of the following codes: 1. A friendly atmosphere with trust 2. creating a happy and dynamic atmosphere 3. Being energetic 4. Non-discrimination and prejudice 5. Flexibility 6. Empathy with the student or the ***"Having pedagogical skills"*** subcategory consists of primary codes: 1. Good presentation and speaking skills 2. Class management 3. Engaging students 4. Getting feedback from students 5. Having academic competence 6. Rewarding participation

System factors are mentioned and it is expected that it will have sub-layers as a layer. Based on the evidence of this study and personal experience, I would like to say that system factors can include two sub-categories, including student-related factors and teacher-related factors, and the codes related to each of these sub-categories can be educational support and support. technical support related to the student as well as educational support and technical support related to the teacher and according to experience and articles studied with the same analysis method, a category cannot end with one theme. In the interviews, the need to support the instructors has yet to be mentioned. If the professors were interviewed, according to your comment, this would be mentioned.

In the method, it’s noted medical students, including doctors, dentists, etc., were interviewed, while only one nurse and midwife was interviewed, and the rest of the students were related to e-learning. Therefore, it is better to mention it as a limitation in generalizing the findings. Based on the results and limitations, suggestions for further studies should be provided. In qualitative research, generalizability is not considered. However, according to the valuable comment of the honorable reviewer, sampling with maximum variation is necessary. Due to the general nature of their education (blended learning), most of the students interviewed were students of virtual colleges who often had a background in medicine-related fields. Virtualization of education in all educational institutions, students of one of the universities of medical sciences were selected as samples. The selected participants from the desired university of medical sciences included a Nursing student, a Midwifery student, and a Dental student.

On the other hand, due to the coincidence of the study with the COVID pandemic, the sampling was first started from the selected University of Medical Sciences. Due to the sudden transition from face-to-face to virtual education and to gain richer experiences for the participants, the following students of the two virtual faculties were considered key informants. The critical point is that some students simultaneously studied in medical sciences universities as virtual colleges.

---------------------------------------------------------------------------------------------------------------------

Reviewer #3: A valuable manuscript on teaching and learning. The background and methods were explained well.
Abstract:
Consider using more specific terms when describing the research methods. For example, you mentioned "electronic interviews," which could be clarified as "online interviews" or "virtual interviews."

Various terms are used, such as online, digital, virtual, and e-interview. In specialized texts and articles, the term electronic interview is also standard. Please refer to the following reference. However, according to the reviewer's opinion, the word online was replaced(P:8).

Reference: Parvaresh M, Varaei Sh(2018). Electronic interview in qualitative research. Iranian Nursing and Midwifery Journal. 31(112):1-5.

It might be beneficial to include a brief sentence highlighting the main practical implications of your study. What can educators, institutions, or policymakers learn from your findings.

please write e- learning in the keywords. It was added(P:3).

Introduction:
you've mentioned that "the quality of interpersonal interactions is a neglected link in e-learning," you could expand on this by briefly discussing why this gap in research exists and why it's important to fill it. It was corrected (P: 6).

Carefully review the grammar, especially in longer sentences. It was corrected.

Methods:
Please provide a sufficient explanation about the field of study and students in the methods section. It was added(P:7-8).

Results:
In the result section, the third theme can emerge into the second one.

The second theme is about the characteristics of formal and informal platforms for providing virtual education and the characteristics related to each. However, the third theme concerns the need to support learners to increase the quality of interpersonal interactions. These two are separate and unrelated. Should they still be merged?

Conclusion:
It would be good if the author could explain the practical implication of this study. It was added(P:22).

……………………………………………………………………………………………………

Reviewer #4: The study suffers from some pitfalls. They are listed below:

- The introduction is chaotic. It does not mirror the significance of the study. It was corrected(P:4-7).
- The study lacks a rigorous literature review to explain the key concepts of the study. It was added, and highlighted(P:22).

- The design of the study is not clear. Explain why the authors have used this design. The study's design (qualitative research of the content analysis type) is mentioned. The reason for choosing it is explained below. However, additional clarifications were added and highlighted(P:7).

***The qualitative descriptive design via conventional content analysis was used to explore students' experiences of interpersonal interaction quality in e-learning. This type of design is usually appropriate when the existing theory or research literature about a phenomenon needs to be revised***.

- The participants section needs revisions to include more demographic information about the participants. Moreover, the author should explain how they accessed the participants and earned their consent. Regarding the participants' demographic information, according to the sampling method (purposive and with maximum variation), we tried to complete the necessary information including the following: The desired theme label was modified. Gender, Age (year), Previous study, Marital status, University, Field of study, Semester, Degree.

One of the crucial challenges of electronic and online interviews is ethical considerations and obtaining consent from participants. For this purpose, the informed consent form was sent to the participants one day before the interview through email or social networks so that they had enough time to read it. The interview was conducted after obtaining consent and signing and sending the consent form to the researcher. This phrase was added in the text.

- The procedures taken for data analysis should be expanded and verified. The data analysis procedures should be clearly mentioned and defined for readers. It was corrected, and highlighted(P:8-9).

- Enough information about the results should be provided for readers. It was corrected(P:9-16).

- The qualitative results need to be revised. First present the theme, then define it, after that offer some excerpts to support the themes. It was corrected(P:9-16).

- The discussion section is really poor. It does not discuss the findings critically. The discussion was revised(P:16-21).

- The study does not have suggestions for further research. It was added(P:21).

- The study lacks implications for the pertinent stakeholders. Please fix this problem. It was fixed(P:22).

- The references are not following APA guidelines. Please revise them. PLOS uses “Vancouver” style, as outlined in the [ICMJE sample references](http://www.nlm.nih.gov/bsd/uniform_requirements.html). (Guideline for authors)

With best regards,

6. PLOS authors have the option to publish the peer review history of their article ([what does this mean?](https://journals.plos.org/plosone/s/editorial-and-peer-review-process#loc-peer-review-history)). If published, this will include your full peer review and any attached files.


If you choose “no”, your identity will remain anonymous but your review may still be made public.


**Do you want your identity to be public for this peer review?** For information about this choice, including consent withdrawal, please see our [Privacy Policy](https://www.plos.org/privacy-policy).

Reviewer #1: **Yes:** Shadi Asadzandi

Reviewer #2: No

Reviewer #3: **Yes:** Mitra Amini

Reviewer #4: No

[NOTE: If reviewer comments were submitted as an attachment file, they will be attached to this email and accessible via the submission site. Please log into your account, locate the manuscript record, and check for the action link "View Attachments". If this link does not appear, there are no attachment files.]

While revising your submission, please upload your figure files to the Preflight Analysis and Conversion Engine (PACE) digital diagnostic tool, <https://pacev2.apexcovantage.com/>. PACE helps ensure that figures meet PLOS requirements. To use PACE, you must first register as a user. Registration is free. Then, login and navigate to the UPLOAD tab, where you will find detailed instructions on how to use the tool. If you encounter any issues or have any questions when using PACE, please email PLOS at [figures@plos.org](mailto:figures@plos.org). Please note that Supporting Information files do not need this step.
