# Supplementary material for: Students’ experience of interpersonal interactions quality in e-Learning: A qualitative research
Source: PLoS One. 2024 Mar 26;19(3):e0298079. doi: 10.1371/journal.pone.0298079 (PMC10965068; doi:10.1371/journal.pone.0298079)
Supplement: Attachment — Submitted filename: Response to Reviewers .docx [file pone.0298079.s005.docx]

1. If the authors have adequately addressed your comments raised in a previous round of review and you feel that this manuscript is now acceptable for publication, you may indicate that here to bypass the “Comments to the Author” section, enter your conflict of interest statement in the “Confidential to Editor” section, and submit your "Accept" recommendation.

Reviewer #1: All comments have been addressed

Reviewer #2: All comments have been addressed

Reviewer #3: All comments have been addressed

Reviewer #4: (No Response)

Reviewer #5: All comments have been addressed

2. Is the manuscript technically sound, and do the data support the conclusions?

The manuscript must describe a technically sound piece of scientific research with data that supports the conclusions. Experiments must have been conducted rigorously, with appropriate controls, replication, and sample sizes. The conclusions must be drawn appropriately based on the data presented.

Reviewer #1: Yes

Reviewer #2: Yes

Reviewer #3: Yes

Reviewer #4: Partly

Reviewer #5: Yes

3. Has the statistical analysis been performed appropriately and rigorously?

Reviewer #1: Yes

Reviewer #2: N/A

Reviewer #3: Yes

Reviewer #4: No

Reviewer #5: Yes

4. Have the authors made all data underlying the findings in their manuscript fully available?

The [PLOS Data policy](http://www.plosone.org/static/policies.action#sharing) requires authors to make all data underlying the findings described in their manuscript fully available without restriction, with rare exception (please refer to the Data Availability Statement in the manuscript PDF file). The data should be provided as part of the manuscript or its supporting information, or deposited to a public repository. For example, in addition to summary statistics, the data points behind means, medians and variance measures should be available. If there are restrictions on publicly sharing data—e.g. participant privacy or use of data from a third party—those must be specified.

Reviewer #1: Yes

Reviewer #2: Yes

Reviewer #3: Yes

Reviewer #4: Yes

Reviewer #5: Yes

5. Is the manuscript presented in an intelligible fashion and written in standard English?

PLOS ONE does not copyedit accepted manuscripts, so the language in submitted articles must be clear, correct, and unambiguous. Any typographical or grammatical errors should be corrected at revision, so please note any specific errors here. It has been corrected. Phrases and words are highlighted in red.

Reviewer #1: Yes

Reviewer #2: Yes

Reviewer #3: Yes

Reviewer #4: No

Reviewer #5: Yes

6. Review Comments to the Author

Please use the space provided to explain your answers to the questions above. You may also include additional comments for the author, including concerns about dual publication, research ethics, or publication ethics. (Please upload your review as an attachment if it exceeds 20,000 characters)

Reviewer #1: All my comments have been applied in the article and I think the article has no problem and has been accepted

Reviewer #2: (No Response)

Reviewer #3: Dear authors
Thanks for revision. The manuscript is now acceptable. You answered all of my comments.

Reviewer #4: Though the authors made some modifications, there are still major issues with the manuscript. I just listed some of them below:

- There are many claims in the introduction part which need citations. Please fix this problem. ***All mentioned phrases are cited in the text of the introduction. Again, the background and review of the literature were revised.***

- The citations are not following the format of the journal. Please fix this problem. ***They were corrected.***

- The significance of the study has not been well discussed in the introduction parts. It is not clear why the authors decided to conduct this study. ***Done***

- The theoretical background of the study is missing. The authors are supposed to explain the theoretical underpinning on which the study has been rested. ***Done***

- The required information about the participants should be discussed. ***It has been mentioned.*** (P:9)

Moreover, the authors are supposed to explain how they met the ethical requirements during the study. ***It has been mentioned.*** (P:9,11)

- The data collection procedures are quiet vague. Please rephrase this part. ***It was corrected.***

- The procedures taken to analyses the gained data are quiet problematic. The authors should explain in details the ways through which they might have analyzed the data***. Thanks for your valuable comment. In the statistical analysis section, we talked about the data analysis method in detail. At first, the data analysis method and its steps are described. In the following, we have said what we have done at each step. We added some explanations again. I hope it is clear and clears our confusion. The method of data analysis is well-known in health care research and is frequently used by researchers in qualitative content analysis. The reference is also introduced. We used Graneheim and Lundman (2004) method.***

Additionally, it should be reported the ways through which the authors measured the reliability and validity of the gained findings. ***Thanks for your comments. The validity and reliability of the data are mentioned with its equivalent term in qualitative research called Trustworthiness and based on the 4 criteria of Lincoln and Guba in the methodology section. Coding was done only by one of the authors, and there was no need to calculate the agreement between the coders or the intercoder rater.***

- For the finding’s sections, follow the following blueprint:

Theme …. Definition of Them …. Excerpts support the theme: ***According to your valuable comment, they were corrected.***

- The discussion part needs to be critical. The authors need to revise it to make it more critical based on the available literature. ***It was corrected.***

- Given the limitations imposed on the study, some suggestions for further research need to be offered for potential readers. ***It was mentioned and highlighted (P:23)***

- The references are not following the APA 7th style. ***PLOS uses “Vancouver” style, as outlined in the ICMJE sample references. (*** ***https://journals.plos.org/plosone/s/submission-guidelines)***

- The language of the manuscript needs substantial improvement. There are grammatical and lexical problems throughout the manuscript. ***It was corrected. It was corrected. All phrases and words in the text of the paper are marked in red.***
With best regards,

Reviewer #5: Dear authors,
I had the chance to review the revised version of the paper "Students' Experience of Interpersonal Interactions Quality in e-Learning: A Qualitative Research". I provided a detailed review/evaluation referring to each comment of the previous reviewers with the correction made by the authors in the manuscript. In my overall opinion, the revised version has met the standards of the PLOS ONE journal and could be considered for publication.
Best regards,

7. PLOS authors have the option to publish the peer review history of their article ([what does this mean?](https://journals.plos.org/plosone/s/editorial-and-peer-review-process#loc-peer-review-history)). If published, this will include your full peer review and any attached files.


If you choose “no”, your identity will remain anonymous but your review may still be made public.


**Do you want your identity to be public for this peer review?** For information about this choice, including consent withdrawal, please see our [Privacy Policy](https://www.plos.org/privacy-policy).

Reviewer #1: No

Reviewer #2: No

Reviewer #3: **Yes:** Mitra Amini

Reviewer #4: **Yes:** Afsheen Rezai

Reviewer #5: No

Reviewer #5

2. The paper needs careful proofreading. It suffers from language problems, grammar, repetitions, tenses, subject-verb disagreement, etc). The whole of the paper was reworked (P:4-7). My comment on this correction: *The quality of writing sounded ok to me. Still, in the revised version, there are some sentences which are written in cohesively. But, overall, I would give it a passing score for publication as the structure of the writing does not interfere with the fluency of the text. It is ok.*

***Thanks.***

The study lacks a rigorous literature review to explain the key concepts of the study. It was added, and highlighted(P:22). My comment on this correction: The revised literature shows the conceptual framework and illustrates some empirical studies. This qualifies it for an acceptable literature section for publication in my opinion. Although adding some evaluations of the previous research from a critical perspective would have made it more convincing.

***It was corrected***

The design of the study is not clear. Explain why the authors have used this design. The study's design (qualitative research of the content analysis type) is mentioned. The reason for choosing it is explained below. However, additional clarifications were added and highlighted(P:7). My comment on this correction: The research design is fine; however, the reasons for choosing such a design is missing from the text. Overall it is ok now.

***It was corrected and highlighted.***

- The discussion section is really poor. It does not discuss the findings critically. The discussion was revised(P:16-21). My comment on this correction: The revised version is much better; however, adding some elevations from a critical perspective would increase the quality of the paper.

***It was corrected and highlighted.***

The study does not have suggestions for further research. It was added(P:21). My comment on this correction: In my humble opinion, the suggestions for further studies is very poor and is confined only to suggestions for conducting in various contexts. I believe it is fine for meeting a passing score for publication but it is definitely not enough!

***It was corrected and highlighted.***
